# Gut microbiota and oral cavity cancer: a two-sample bidirectional Mendelian randomization study

**DOI:** 10.3389/fonc.2024.1389678

**Published:** 2024-05-30

**Authors:** Zhijuan Sun, Chunying Bai, Dandan Hao, Xiling Jiang, Jianxing Chen

**Affiliations:** ^1^ International Education School, Chifeng University, Chifeng, China; ^2^ School of Basic Medical Sciences, Chifeng University, Chifeng, China; ^3^ Department of Stomatology, Affiliated Hospital of Chifeng University, Chifeng, China; ^4^ College of Agriculture, Chifeng University, Chifeng, China

**Keywords:** causality, gut microbiota, GWAS, Mendelian randomization, oral cavity cancer

## Abstract

This study employs a two-sample bidirectional Mendelian randomization (MR) approach to systematically evaluate the causal relationship between gut microbiota and oral cavity cancer (OCC).

**Objective:**

To address the challenge in establishing the causal relationship between gut microbiota and OCC, we applied a systematic MR analysis.

**Methods:**

Utilizing GWAS data from the MiBioGen consortium (18,340 individuals) and UK Biobank (n = 264,137), we selected instrumental variables and employed MR-Egger, weighted median, IVW, and weighted mode analyses. Heterogeneity and pleiotropy were assessed using Cochran’s Q test and MR-Egger intercept test.

**Results:**

Our findings indicate, at the order level, *Bacteroidales* (OR = 0.9990, 95% CI = 0.9980–1.0000, *P* = *0.046*), *Burkholderiales* (OR = 1.0009, 95% CI = 1.0001–1.0018, *P* = *0.033*), and *Victivallales* (OR = 0.9979, 95% CI = 0.9962–0.9995, *P* = *0.037*) exhibit causality on OCC in the Weighted median, IVW, and MR-Egger analyses, respectively. At the family level, *Alcaligenaceae* (OR = 1.0012, 95% CI = 1.0004–1.0019, *P* = *0.002*) and *Clostridiaceae1* (OR = 0.9970, 95% CI = 0.9948–0.9992, *P* = *0.027*) show causality on OCC in IVW and MR-Egger analyses. At the genus level, *Clostridiumsensustricto1* (IVW, OR = 0.9987, 95% CI = 0.9980–0.9995, *P* = *0.001*; MR-Egger, OR = 0.9978, 95% CI = 0.9962–0.9995, *P* = *0.035*), *Desulfovibrio* (IVW, OR = 1.0008, 95% CI = 1.0001–1.0015, *P* = *0.016*), *Eggerthella* (IVW, OR = 0.9995, 95% CI = 0.9990–1.0000, *P* = *0.048*), *Eubacterium fissicatena* group (IVW, OR = 1.0005, 95% CI = 1.0000–1.0009, *P* = *0.032*), and *Holdemanella* (IVW, OR = 0.9994, 95% CI = 0.9989–0.9999, *P* = *0.018*) are implicated in causing OCC in related analyses.

**Conclusion:**

Our study identifies *Burkholderiales* order, *Alcaligenaceae* family, *Desulfovibrio* genus, and *Eubacterium fissicatena* group as causally increasing OCC risk. In contrast, *Bacteroidales* order, *Victivallales* order, *Clostridiaceae1* family, *Clostridiumsensustricto1* genus, *Eggerthella* genus, and *Holdemanella* genus are causally associated with a decreased OCC risk. However, further investigations are essential to delineate an optimal gut microbiota composition and unravel the underlying mechanisms of specific bacterial taxa in OCC pathophysiology.

## 1 Introduction

Oral cavity cancer (OCC) stands as a substantial global health concern, particularly marked by its varied incidence rates across regions, highlighting the disease’s complex and multifaceted nature. With over 377,000 new cases annually, OCC, predominantly Oral squamous cell carcinoma (OSCC), represents the 16th most prevalent cancer globally and affects males, representing 70% of reported cases. Regional disparities in incidence, with Melanesia and South-Central Asia displaying the highest rates, underscore the multifaceted nature of this malignancy (1). In the United States, OCC accounts for 52,010 new cases annually, comprising 4% of all cancer incidences. While the European Union records approximately 128,600 new cases of head and neck cancer, which includes OCC, the significance of these numbers lies in the potential for region-specific preventive strategies. India, with over 1,000,000 squamous cell carcinoma cases annually, presents a unique context for studying OCC due to its high incidence and the role of cultural practices, such as tobacco and areca nut use, which are known carcinogens in the region. Taiwan reported 8,204 cases of squamous cell carcinoma of the head and neck in 2019, ranking as the fifth leading cause of cancer death (2).

Understanding the underlying causes of OCC is crucial for developing effective preventive measures. While tobacco remains a primary contributor, certain geographic areas highlight areca nut as a significant carcinogen (1), and human papillomavirus (HPV) has been established as crucial in oropharyngeal SCC but not in OSCC (3, 4), adds to the disease’s complexity. Discrepancies arise from diverse factors such as tobacco and alcohol usage, dietary practices, oral hygiene, socioeconomic status, and HPV exposure (3, 4).

Primarily affecting critical areas within the oral cavity, OCC encompasses various subtypes, including OSCC, adenocarcinoma, mucoepidermoid carcinoma, and occasionally, malignant melanomas. OSCC often arises from pre-existing oral epithelial dysplasia (OED), marked by high mutational loads and gross chromosomal changes (4). Chromosomal instability escalation correlates with an increased likelihood of OSCC development. Given these circumstances, understanding the underlying causes and risk factors associated with oral cancer is imperative for developing effective preventive strategies.

The human microbiome, particularly the gut microbiota, has garnered increasing attention due to its association with a spectrum of diseases, including various cancers (5–9). Within the human gut, a diverse ecosystem thrives, housing an array of microbes comprising bacteria, archaea, eukarya, viruses, and parasites, with bacteria prevailing as the dominant population (10). Dysbiosis within the gut microbiota has been implicated in the pathogenesis of a myriad of human disorders, ranging from inflammatory bowel disease to Alzheimer’s disease and various cancers. This underscores the pivotal role of the gut microbiota in maintaining health and the potential consequences when this delicate balance is perturbed. As our understanding of the intricate connections between the gut microbiome and cancer deepens, emerging evidence underscores the pivotal role of the gut microbiota in cancer progression and treatment outcomes (11). Illustratively, a study showcased the impact of an oral antibiotics cocktail (4Abx) on OSCC mice, inducing alterations in the gut microbiota and perturbing microbial metabolism. This led to elevated tyrosine levels and reduced glutamate levels, ultimately fostering tumor development (12). Furthermore, epidemiological investigations have delineated distinctions in microbial composition between patients with OSCC and healthy counterparts, revealing diminished bacterial diversity. This observation suggests the potential utility of microbiome biomarkers in oral cancer screening and prognosis (13).

MR emerges as a powerful tool utilizing genetic variants, robustly linked to exposures, as instrumental variables (IVs) to establish causality between a risk factor and a healthy outcome (14, 15). This approach furnishes more robust evidence for causal inference than observational epidemiology by mitigating the influence of potential confounding, reverse causation, and various biases (16, 17). In a recent publication, a comprehensive series of genome-wide association studies (GWASs) delved into the impact of host genetic loci on various intestinal bacterial taxa, providing summary statistics for 211 taxa (18). This facilitated the exploration of potential causal effects of the human gut microbiota on diverse disease outcomes through the MR approach. In our exhaustive review of the literature exploring the interplay between the gut microbiota and OCC, a mounting body of evidence has surfaced, indicating a nuanced relationship. Employing MR techniques, such as inverse variance weighting (IVW) and MR Egger methods, recent studies, including those by Liu et al. (19), Xiang et al. (20), and Zhang et al. (21), have unveiled novel insights into the potential causal effects of gut microbiota on oral cancer risk. These robust findings, supported by rigorous statistical analyses and sensitivity tests, underscore the imperative for further exploration into the mechanisms through which gut microbiota may influence the development of oral cancer. This pursuit holds promise for delineating avenues for future preventive and therapeutic strategies.

While existing MR studies have explored the causal relationship between gut microbiota and OCC (19–21), it’s noteworthy that Liu et al. (19) and Xiang et al. (20) focused on the oral cavity cancer dataset of 2016 (ieu-b-94), and Zhang et al. (21) examined all datasets of Oral Cavity and Pharyngeal Cancer, not the more recent dataset used in our study (ieu-b-4961) from 2021. Our current investigation aims to build upon these findings by employing a more recent and stringent dataset (ieu-b-4961) from the UK Biobank, offering a comprehensive exploration of the causality between gut microbiota and OCC across different taxonomic levels.

In this study, we not only explore the different taxonomic levels (phylum, class, order, family, and genus) of bacterial taxa and leveraging updated GWAS summary data for OCC with a stringent new dataset, but also conduct MR analyses in the reverse direction to discern any potential causal effects of OCC on gut microbiota. This study holds promise in offering valuable insights into the role of the gut microbiome in OCC, paving the way for future preventive and therapeutic strategies in combating this formidable disease.

## 2 Materials and methods

### 2.1 Data sources for gut microbiota and OCC

The gut microbiota-related GWAS data were procured from the international consortium MiBioGen (22). This consortium conducted a comprehensive, multiethnic, and genome-wide meta-analysis exploring the associations between autosomal human genetic variants and the gut microbiome (23). Inclusion criteria for the participants in the MiBioGen study were clearly defined, including age-appropriate adults from diverse ethnic backgrounds, without specific exclusion for health conditions, thus providing a broad representation of the general population. For the control group in our study, we strictly adhered to the inclusion criteria that ensured participants were healthy, free from any known chronic diseases, and representative of the general population across various demographic factors. Encompassing 18,340 participants from 24 cohorts spanning the USA, Canada, Israel, South Korea, Germany, Denmark, the Netherlands, Belgium, Sweden, Finland, and the UK, the meta-analysis provided a robust foundation for our study.

Upon adjusting for age, sex, technical covariates, and genetic principal components, the quantitative microbiome trait loci (mbQTL) analysis in the MiBioGen study yielded 211 microbial taxa-related GWAS summary statistics. These encompassed 9 phyla, 16 classes, 20 orders, 35 families (including 3 unknown families), and 131 genera (including 12 unknown genera). Further details regarding the microbiota data are available in the original study by Brown et al. (23).

In our investigation, our focus primarily centered on MR analyses spanning from the phylum level to the genus level. For taxonomic categorization, we utilized the Taxonomy Browser tool on NCBI (https://www.ncbi.nlm.nih.gov/guide/taxonomy/) to navigate the taxonomy tree for each microbial taxon. Detailed categorization specifics are documented in the work of Luo et al. (24).

The summary statistics data for oral cavity cancer (OCC), consisting of 357 cases and 372,016 controls, were sourced from the latest release of the UK Biobank consortium in March 2022, identified as Dataset: ieu-b-4961. Notably, ethical approval for this study was deemed unnecessary, as the original studies providing the data had already secured appropriate ethics and institutional review board approval.

### 2.2 Assumptions of the MR study

**Figure 1:**
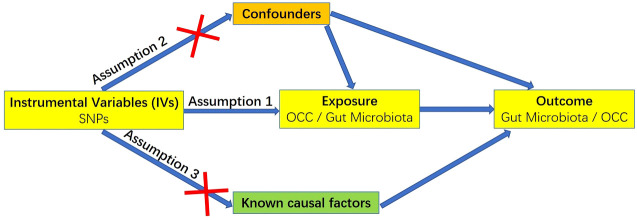
Three key assumptions for a valid Mendelian randomization study.

A MR study relies on three fundamental assumptions, illustrated in **Figure 1** (25). Firstly, the chosen genetic variants employed as IVs must exhibit a robust association with the target exposure. The strength of these instruments is often assessed using the F statistic, computed as F = R²(n-k-1)/k(1-R²), where R² denotes the proportion of variance explained by the instruments, n represents the sample size, and k is the count of selected IVs. To mitigate potential biases stemming from weak instruments, a conventional threshold value of F statistic > 10 was applied in this study (26). Secondly, the absence of unmeasured confounders is crucial for establishing associations between genetic variants and outcomes. Thirdly, genetic variants should solely impact the outcome through their influence on the targeted exposure, ensuring the absence of horizontal pleiotropy in the relationship between genetic variants and outcomes.

### 2.3 Selection and exclusion of instrumental variables

To ascertain the causal impact of the human gut microbiota on OCC, we employed single nucleotide polymorphisms (SNPs) exhibiting associations at p < 1×10–^5^ as instruments in our MR analysis. The inclusion criteria for selecting SNPs as IVs were based on the significance threshold and the ability to serve as strong instruments in the MR analysis, ensuring their reliability in indicating the exposure of interest. This threshold has been identified as optimal in previous gut microbiota-related MR research, maximizing genetic variance explained by predictors and facilitating sensitivity analysis (23, 27).

Subsequently, independent SNPs were chosen based on linkage disequilibrium [LD] r² < 0.001 within a 10,000-kb window using the clumping procedure in 1000 Genomes European (EUR) data within the TwoSampleMR package. In cases where no shared SNP was found between exposure and outcome GWAS data, a proxy SNP with r² > 0.8 was selected as a replacement. Following the retrieval of IVs from OCC GWAS data, SNPs significantly associated with OCC (P outcome < P exposure) were removed during the harmonization process. F statistics were computed for each SNP before harmonization, with SNPs exhibiting F statistics less than 10 considered as weak instruments and subsequently excluded.

To detect and eliminate outlier instruments, we conducted MR Pleiotropy Residual Sum and Outlier (MR-PRESSO) tests. These tests are particularly effective when horizontal pleiotropy is observed in less than 50% of the instruments (28). Only SNPs that passed this rigorous filtering process were eligible for subsequent MR analysis.

**Figure 2:**
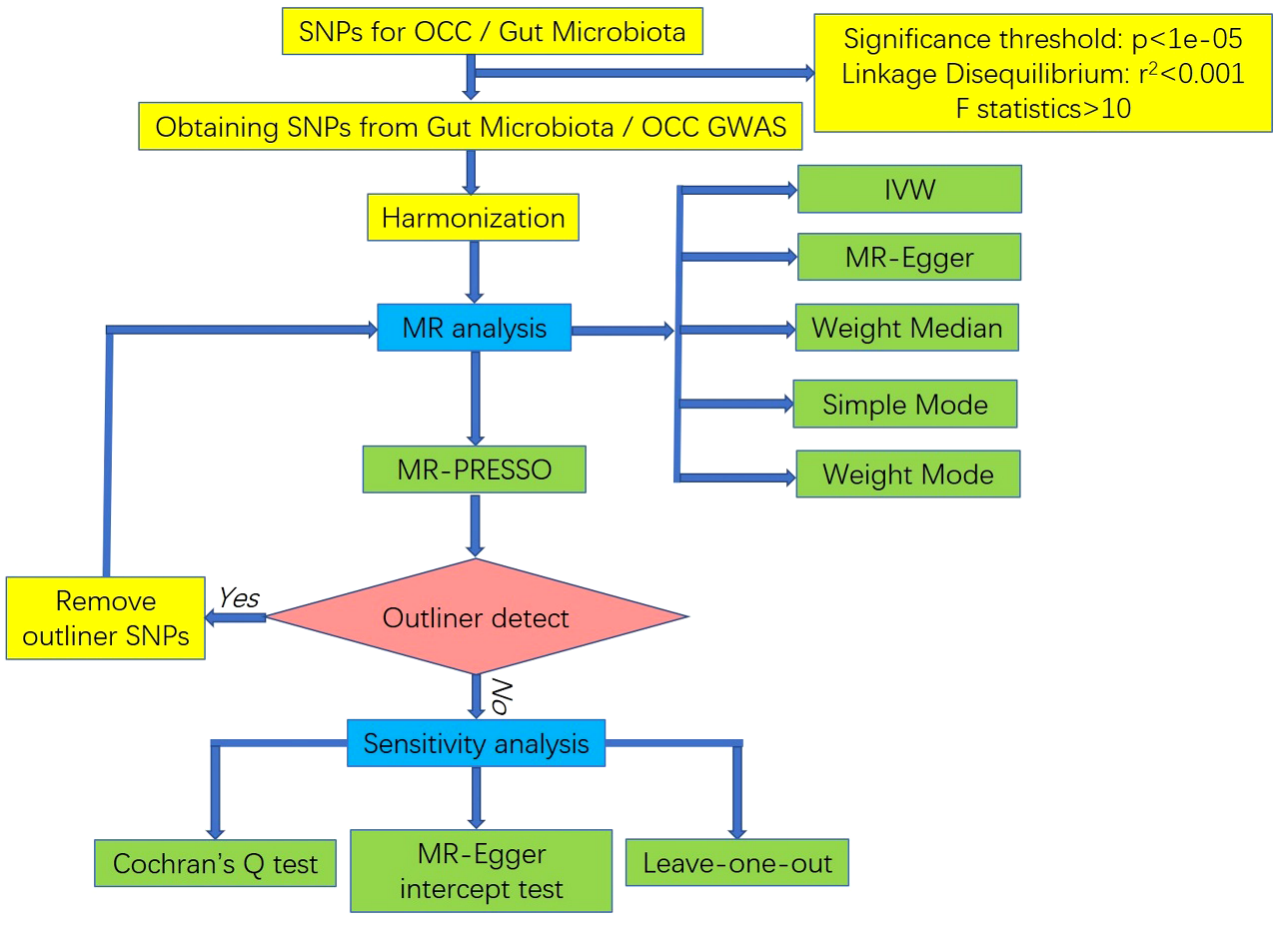
The flowchart of the MR study revealing the causal relationship between gut microbiota and OCC.

In reverse MR analysis, assessing whether OCC influenced microbiome composition, we selected genetic variants associated with OCC at the genome-wide significance level (P < 5×10–^8^) as instruments. The subsequent filtering steps mirrored those applied in the initial selection process. The study’s flowchart is depicted in **Figure 2**.

#### 2.3.1 Controls’ Clinical Characteristics

The control group for the OCC data, consisting of 372,016 individuals, was derived from the UK Biobank consortium. To better specify the clinical characteristics of controls, we ensured that this group was well-characterized in terms of age, sex, ethnicity, and vital health metrics, including information on lifestyle factors such as smoking status, alcohol consumption, and physical activity levels. This comprehensive data allowed for a thorough comparison with the OCC cases.

### 2.4 Mendelian randomization analysis

We conducted MR analysis using five distinct approaches [MR-Egger, weighted median, random-effect inverse variance weighted (IVW), simple mode, weighted mode] to quantify causal relationships between gut microbiota composition and the risk of OCC. The inclusion criteria for the MR analysis were stringent, requiring that each method be applied to a set of IVs that passed the quality control measures, including the F statistic threshold and MR-PRESSO tests for pleiotropy. MR-Egger: Provides a consistent causal effect estimate even in the presence of pleiotropic effects, as long as the association of each genetic variant with the exposure remains independent of pleiotropic effects (29). Weighted Median: Requires that at least 50% of the weight in the analysis originates from variants serving as valid instruments (30). IVW: Functions as a meta-analysis method based on the assumption that instruments exclusively influence the outcome through the exposure of interest and not through any alternative pathway (31). Simple Mode: An approach to estimate the causal effect of a genetic instrument on a specific outcome by averaging effect sizes across different genetic variants associated with the exposure. This method is particularly valuable when multiple genetic variants influence the exposure, aiming to comprehend the overall effect on the outcome (32). Weighted Mode: Remains consistent when the largest subset of instruments identifying the same causal effect are valid instruments, even if the majority of others are invalid (33). Causal relationships were deemed significant when any of these five methods in the MR analysis yielded a P value < 0.05.

Sensitivity analysis was conducted for significant estimates to identify potential heterogeneity and pleiotropy. Heterogeneity was identified using Cochran’s Q test, while the MREgger intercept test assessed horizontal pleiotropy. An insignificant P value (P > 0.05) indicated the absence of heterogeneity or pleiotropy. Additionally, leave-one-out analyses were applied to verify potential bias or influence by a single SNP by iteratively removing each instrumental SNP for repeated IVW analyses.

All analyses were performed using R (version 4.2.1), utilizing the R packages “TwoSampleMR” (version 0.5.6) and “stats” (version 4.2.1). All tests considered results with P values < 0.05 as significant.

This study is reported following the Strengthening the Reporting of Observational Studies in Epidemiology Using Mendelian Randomization guidelines (STROBE-MR, S1 Checklist) (34).

## 3 Results

Our study explores the relationship between the gut microbiota and oral cavity cancer (OCC) by using Mendelian randomization method. Through a careful examination, we identified specific genetic markers, or instrumental variables (IVs), associated with various gut bacteria. These IVs were strong indicators of the presence of these bacteria, which helped us avoid biases in our study. We found that out of the many types of gut bacteria we looked at, only a few were significantly linked to OCC. It’s important to note that for some gut bacteria, we detected ‘horizontal pleiotropy’, which means they may affect OCC through multiple biological pathways. This finding adds complexity to our understanding of how gut microbiota may influence OCC. In essence, our research provides evidence that certain gut bacteria may play a role in the development of oral cavity cancer, offering new insights that could be valuable for future cancer prevention and treatment strategies.

### 3.1 Comprehensive examination of IVs across gut microbiota

Through employing meticulous methodologies, including stringent filtering based on a genome-wide significance threshold (*P* < 1.0 × 10–^5^), rigorous linkage disequilibrium (LD) tests, precise harmonization techniques, and thorough verification of F-statistics, a multitude of SNPs has been identified as instrumental variables for each of the 211 bacterial taxa under investigation. Throughout all analyses, F-statistics were consistently calculated for each retained SNP, all surpassing the threshold value of 10. This robustly indicates substantial correlation strength between the instrumental variables and their corresponding bacterial taxa, establishing them as strong instruments (35). Consequently, our study unequivocally demonstrates its adeptness in circumventing potential biases stemming from weakened instrumental variables.

### 3.2 Selection of instrumental variables from phylum to genus levels

The selection of appropriate instrumental variables (IVs) is a critical step in MR studies, as they serve as proxies for the exposure of interest while meeting specific criteria to ensure a valid causal inference. In the context of our study, we aimed to identify genetic variants, specifically SNPs, that are robustly associated with different taxonomic levels of gut microbiota, from phylum to genus. These SNPs were meticulously chosen to serve as IVs in our MR analysis to assess the potential causal relationship between gut microbiota and OCC.

Our approach to IVs selection was systematic and multi-tiered, involving stringent significance thresholds, clumping processes to account for linkage disequilibrium, and harmonization of exposure and outcome datasets. This methodical selection process ensured that the identified IVs were both genetically robust and relevant to the taxonomic levels of interest.

#### 3.2.1 Phylum level

At the phylum level, our strategy was to select one index SNP each for *Bacteroidetes* (synonym *Bacteroidota*), *Euryarchaeota*, and *Verrucomicrobia*, to genetically predict these broad categories of gut microbiota. For *Proteobacteria* and *Tenericutes*, we chose two index SNPs each, and for *Lentisphaerae*, three index SNPs were selected to capture the genetic variation within these phyla.

#### 3.2.2 Class level

Moving down the taxonomic hierarchy, we identified one index SNP for *Alphaproteobacteria*, *Bacilli*, *Bacteroidia*, *Coriobacteriia*, *Deltaproteobacteria*, and *Verrucomicrobiae*, respectively. For *Betaproteobacteria*, *Gammaproteobacteria*, and *Mollicutes*, two index SNPs were chosen, while *Lentisphaeria* and *Melainabacteria* were each represented by three index SNPs.

#### 3.2.3 Order level

At the order level, our selection criteria led to the identification of one index SNP for *Bacillales*, *Bacteroidales*, *Coriobacteriales*, *Lactobacillales*, *MollicutesRF9*, *NB1n*, *Pasteurellales*, *Rhodospirillales*, and *Verrucomicrobiales*. For *Burkholderiales*, two index SNPs were selected, and for *Enterobacteriales*, *Gastranaerophilales*, and *Victivallales*, three index SNPs were chosen.

#### 3.2.4 Family level

Family-level IV selection was similarly systematic, with one index SNP chosen for *Bacteroidaceae*, *Clostridiaceae1*, *Coriobacteriaceae*, *Pasteurellaceae*, *Peptococcaceae*, *Rhodospirillaceae*, *Ruminococcaceae*, *Veillonellaceae*, *Verrucomicrobiaceae*, and *Victivallaceae*. Two index SNPs were selected for *Alcaligenaceae* and *FamilyXI*, while three index SNPs were chosen for *Enterobacteriaceae* and *Rikenellaceae*.

#### 3.2.5 Genus level

Finally, at the genus level, we selected one index SNP for *Adlercreutzia*, *Akkermansia*, *Alistipes*, and so on, through a comprehensive list of genera. For certain genera with greater genetic variability or where more precision was required, two or three index SNPs were chosen.

In summary, our analysis revealed that gut microbiota across 6 phyla, 11 classes, 13 orders, 14 families, and 63 genera exhibited a significant association with the pathogenesis or disease progression of OCC at various taxonomic levels. However, only a subset of these taxa, represented by 1 phylum, 1 class, 3 orders, 2 families, and 5 genera, demonstrated a significant association with OCC when their SNPs were utilized as IVs in MR analysis.

### 3.3 Causal association of gut microbiota classification levels with OCC

To elucidate the causal relationship between human gut microbiota and OCC, we conducted MR analyses encompassing bacterial taxa from the phylum to genus levels.

#### 3.3.1 MR analyses of phylum level gut microbiota with OCC

**Figure 3:**
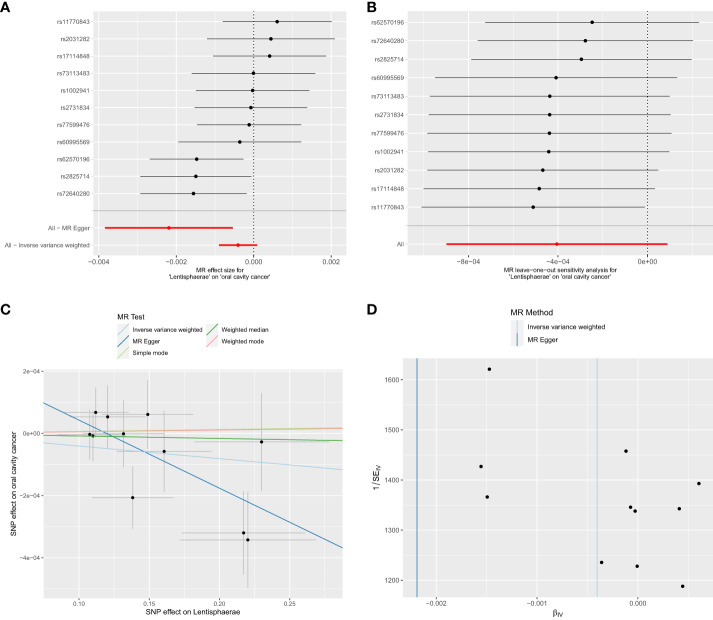
Significant MR estimates of phylum-level gut microbiota (*Lentisphaerae*) on OCC. **(A)** forest plot; **(B)** leave-one-out analysis; **(C)** scatter plot; **(D)** funnel plot.

Out of the 6 selected and retained phyla (*Bacteroidetes* or *Bacteroidota*, *Euryarchaeota*, *Verrucomicrobia*, *Proteobacteria*, *Tenericutes*, and *Lentisphaerae*), only *Lentisphaerae* exhibited a causal association with OCC. Specifically, *Lentisphaerae* demonstrated a significant correlation with OCC (OR = 0.9978, 95% CI = 0.9962–0.9995, P = 0.029) in the MR-Egger analysis. Detailed results are presented in **Table 1** and **Figure 3**.

**Table 1:** Significant and nominally significant MR estimates of the associations between gut microbiota and OCC.

Taxa	Gut Microbiota (exposure)	Trait (outcome)	Nsnp	Methods	Beta	SE	OR (95%CI)	*p*	Heterogeneity		Horizontal pleiotropy		
									Cochran’s Q	*p*	Egger intercept	SE	*p*
**Phylum**	*Lentisphaerae*	OCC	11	MR Egger	-0.0022	0.0008	0.9978(0.9962–0.9995)	***0.029***	8.1374	*0.5204*	0.0003	0.0001	*0.0555*
				Inverse variance weighted	-0.0004	0.0003	0.9996(0.9991–1.0001)	*0.108*					
				Weighted median	-8.01E-05	0.0003	0.9999(0.9993–1.0006)	*0.808*					
				Simple mode	6.42E-05	0.0005	1.0001(0.9990–1.0011)	*0.907*					
				Weighted mode	5.53E-05	0.0005	1.0001(0.9990–1.0011)	*0.920*					
**Class**	*Lentisphaeria*	OCC	10	MR Egger	-0.0022	0.0009	0.9979(0.9962–0.9995)	***0.037***	8.3425	*0.4008*	0.0003	0.0001	*0.0681*
				Inverse variance weighted	-0.0004	0.0003	0.9996(0.9990–1.0001)	*0.140*					
				Weighted median	-6.20E-05	0.0003	0.9999(0.9993–1.0006)	*0.853*					
				Simple mode	0.0001	0.0006	1.0001(0.9990–1.0012)	*0.821*					
				Weighted mode	0.0001	0.0006	1.0001(0.9990–1.0013)	*0.848*					
**Order**	*Bacteroidales*	OCC	14	MR Egger	-0.0013	0.0009	0.9987(0.9969–1.0004)	*0.161*	13.9596	*0.3033*	6.84E-05	6.64E-05	*0.3231*
				Weighted median	-0.0010	0.0005	0.9990(0.9980–1.0000)	***0.046***					
				Inverse variance weighted	-0.0005	0.0004	0.9995(0.9987–1.0002)	*0.190*					
				Simple mode	-0.0003	0.0008	0.9997(0.9981–1.0014)	*0.766*					
				Weighted mode	-0.0008	0.0006	0.9992(0.9979–1.0004)	*0.223*					
**Order**	*Burkholderiales*	OCC	13	MR Egger	0.0009	0.0014	1.0009(0.9981–1.0037)	*0.537*	15.6344	*0.2086*	1.86E-06	9.82E-05	*0.9852*
				Weighted median	0.0003	0.0006	1.0003(0.9992–1.0014)	*0.590*					
				Inverse variance weighted	0.0009	0.0004	1.0009(1.0001–1.0018)	***0.033***					
				Simple mode	-5.40E-05	0.0011	0.9999(0.9978–1.0021)	*0.961*					
				Weighted mode	-0.0001	0.0009	0.9999(0.9980–1.0017)	*0.887*					
**Order**	*Victivallales*	OCC	10	MR Egger	-0.0022	0.0009	0.9979(0.9962–0.9995)	***0.037***	8.3425	*0.4008*	0.0003	0.0001	*0.0681*
				Weighted median	-6.20E-05	0.0003	0.9999(0.9993–1.0006)	*0.853*					
				Inverse variance weighted	-0.0004	0.0003	0.9996(0.9990–1.0001)	*0.140*					
				Simple mode	0.0001	0.0006	1.0001(0.9990–1.0012)	*0.821*					
				Weighted mode	0.0001	0.0005	1.0001(0.9990–1.0012)	*0.838*					
**Family**	*Alcaligenaceae*	OCC	15	Inverse variance weighted	0.0012	0.0004	1.0012(1.0004–1.0019)	***0.002***	15.1577	*0.3675*	-4.63E-05	0.0001	*0.6751*
				Weighted median	0.0010	0.0005	1.0010(1.0000–1.0020)	*0.055*					
				Weighted mode	0.0014	0.0009	1.0014(0.9997–1.0031)	*0.120*					
				Simple mode	0.0013	0.0009	1.0013(0.9996–1.0031)	*0.148*					
				MR Egger	0.0018	0.0015	1.0018(0.9988–1.0048)	*0.263*					
**Family**	*Clostridiaceae1*	OCC	11	MR Egger	-0.0030	0.0011	0.9970(0.9948–0.9992)	***0.027***	3.8597	*0.9204*	0.0002	8.22E-05	*0.0544*
				Inverse variance weighted	-0.0006	0.0004	0.9994(0.9986–1.0001)	*0.099*					
				Weighted median	-0.0006	0.0005	0.9994(0.9984–1.0004)	*0.266*					
				Weighted mode	-0.0008	0.0010	0.9992(0.9972–1.0012)	*0.455*					
				Simple mode	0.0003	0.0009	1.0003(0.9986–1.0020)	*0.743*					
**Genus**	*Clostridiumsensustricto1*	OCC	9	Inverse variance weighted	-0.0013	0.0004	0.9987(0.9980–0.9995)	***0.001***	7.9549	*0.4379*	0.0001	8.44E-05	*0.2665*
				MR Egger	-0.0022	0.0008	0.9978(0.9962-0.9995)	***0.035***					
				Weighted median	-0.0010	0.0005	0.9990(0.9979-1.0000)	*0.061*					
				Weighted mode	-0.0011	0.0007	0.9989(0.9976-1.0003)	*0.154*					
				Simple mode	-0.0010	0.0008	0.9990(0.9975-1.0005)	*0.212*					
**Genus**	*Desulfovibrio*	OCC	10	Inverse variance weighted	0.0008	0.0003	1.0008(1.0001-1.0015)	***0.016***	9.6202	*0.3821*	4.89E-05	0.0001	*0.6447*
				Weighted median	0.0006	0.0005	1.0006(0.9997-1.0015)	*0.174*					
				Weighted mode	0.0004	0.0007	1.0004(0.9992-1.0017)	*0.514*					
				Simple mode	0.0004	0.0007	1.0004(0.9990-1.0019)	*0.560*					
				MR Egger	0.0003	0.0010	1.0003(0.9983-1.0024)	*0.749*					
**Genus**	*Eggerthella*	OCC	9	Inverse variance weighted	-0.0005	0.0003	0.9995(0.9990–1.0000)	***0.048***	4.9147	*0.7666*	-9.54E-06	0.0001	*0.9420*
				Weighted median	-0.0005	0.0004	0.9995(0.9988–1.0002)	*0.135*					
				Simple mode	-0.0005	0.0005	0.9995(0.9984–1.0005)	*0.355*					
				Weighted mode	-0.0005	0.0005	0.9995(0.9984–1.0006)	*0.376*					
				MR Egger	-0.0004	0.0011	0.9996(0.9973–1.0018)	*0.713*					
**Genus**	*Eubacterium fissicatena* group	OCC	9	Inverse variance weighted	0.0005	0.0002	1.0005(1.0000-1.0009)	***0.032***	4.9953	*0.7581*	-7.06E-06	0.0002	*0.9654*
				Weighted median	0.0003	0.0003	1.0003(0.9997-1.0009)	*0.347*					
				Simple mode	0.0003	0.0005	1.0003(0.9993-1.0012)	*0.582*					
				Weighted mode	0.0003	0.0005	1.0003(0.9994-1.0012)	*0.582*					
				MR Egger	0.0005	0.0012	1.0005(0.9982-1.0029)	*0.667*					
**Genus**	*Holdemanella*	OCC	13	Inverse variance weighted	-0.0006	0.0002	0.9994(0.9989–0.9999)	***0.018***	11.5196	*0.4850*	-4.37 E-05	8.00E-05	*0.5962*
				Weighted median	-0.0004	0.0003	0.9996(0.9989–1.0002)	*0.217*					
				Weighted mode	-0.0003	0.0005	0.9998(0.9987–1.0008)	*0.641*					
				Simple mode	-0.0002	0.0006	0.9998(0.9987–1.0009)	*0.714*					
				MR Egger	-0.0002	0.0008	0.9998(0.9983–1.0013)	*0.809*					

Nsnp, Number of SNP; Beta, Beta coefficient; SE, Standard Error; OR, Odds Ratio.p-values < 0.05 (in bold) denote statistical significance.

#### 3.3.2 MR analyses of class-level gut microbiota with OCC

**Figure 4:**
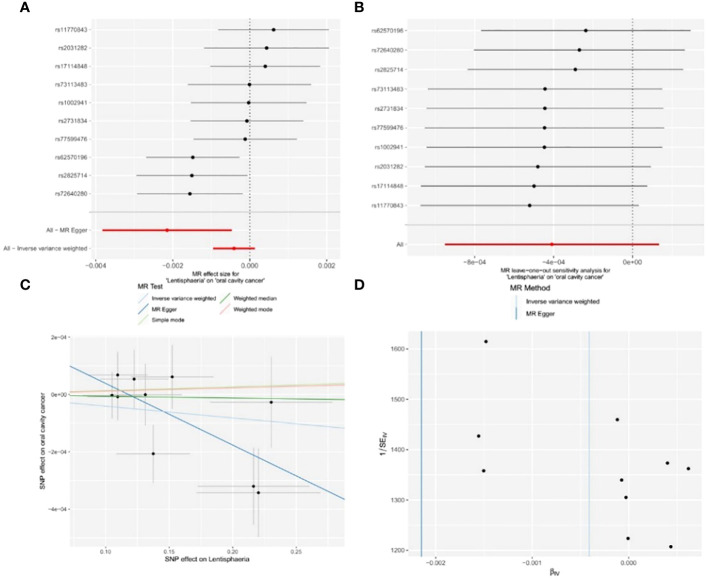
Significant MR estimates of class-level gut microbiota (*Lentisphaeria*) on OCC. **(A)** forest plot; **(B)** leave-one-out analysis; **(C)** scatter plot; **(D)** funnel plot.

Out of the 11 retained and selected classes (*Alphaproteobacteria*, *Bacilli*, *Bacteroidia*, *Coriobacteriia*, *Deltaproteobacteria*, *Verrucomicrobiae*, *Betaproteobacteria*, *Gammaproteobacteria*, *Mollicutes*, *Lentisphaeria*, and *Melainabacteria*), only *Lentisphaeria* exhibited a causal association with OCC. Specifically, it demonstrated a significant correlation with OCC (OR = 0.9979, 95% CI = 0.9962–0.9995, P = 0.037) in the MR-Egger analysis. Detailed results are presented in **Table 1** and **Figure 4**.

#### 3.3.3 MR analyses of order-level gut microbiota with OCC

**Figure 5:**
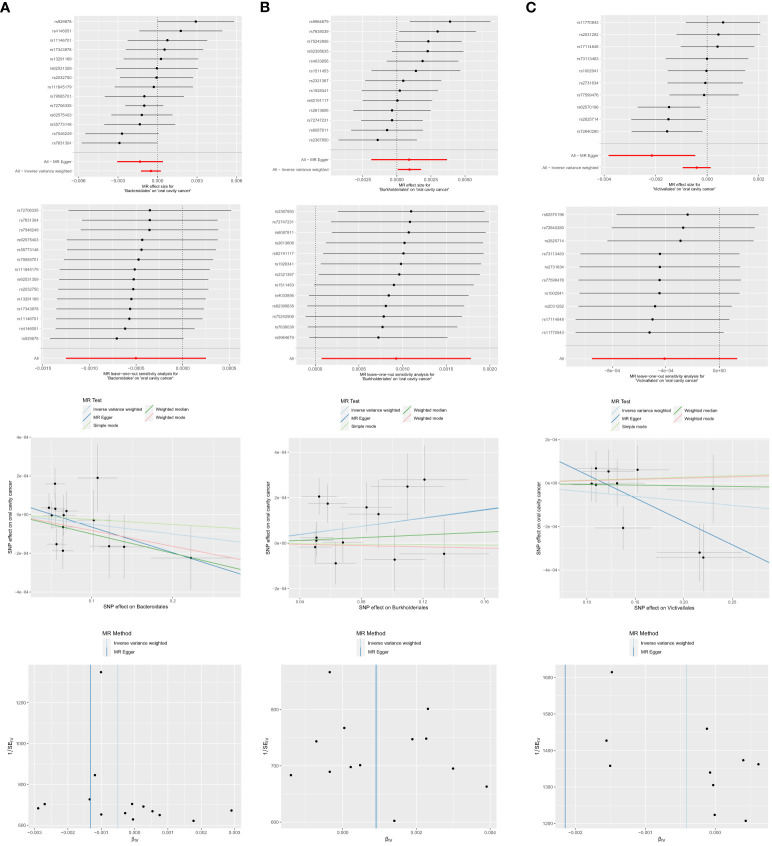
Significant MR estimates of order-level gut microbiota **(A)** *Bacteroidales* **(B)** *Burkholderiales* **(C)** *Victivallales* on OCC. From top to bottom: forest plot; leave-one-out analysis; scatter plot; funnel plot.

Among the 13 retained and selected orderes (*Bacillales*, *Bacteroidales*, *Coriobacteriales*, *Lactobacillales*, *MollicutesRF9*, *NB1n*, *Pasteurellales*, *Rhodospirillales*, *Verrucomicrobiales*, *Burkholderiales*, *Enterobacteriales*, *Gastranaerophilales* and *Victivallales*), *Bacteroidales* (OR = 0.9990, 95% CI = 0.9980–1.0000, P = 0.046), *Burkholderiales* (OR = 1.0009, 95% CI = 1.0001–1.0018, P = 0.033) and *Victivallales* (OR = 0.9979, 95% CI = 0.9962–0.9995, P = 0.037) were indicated to have causality on OCC in the analyses of Weighted median, IVW and MR-Egger, respectively. Detailed results are presented in **Table 1** and **Figure 5**.

#### 3.3.4 MR analyses of family-level gut microbiota with OCC

**Figure 6:**
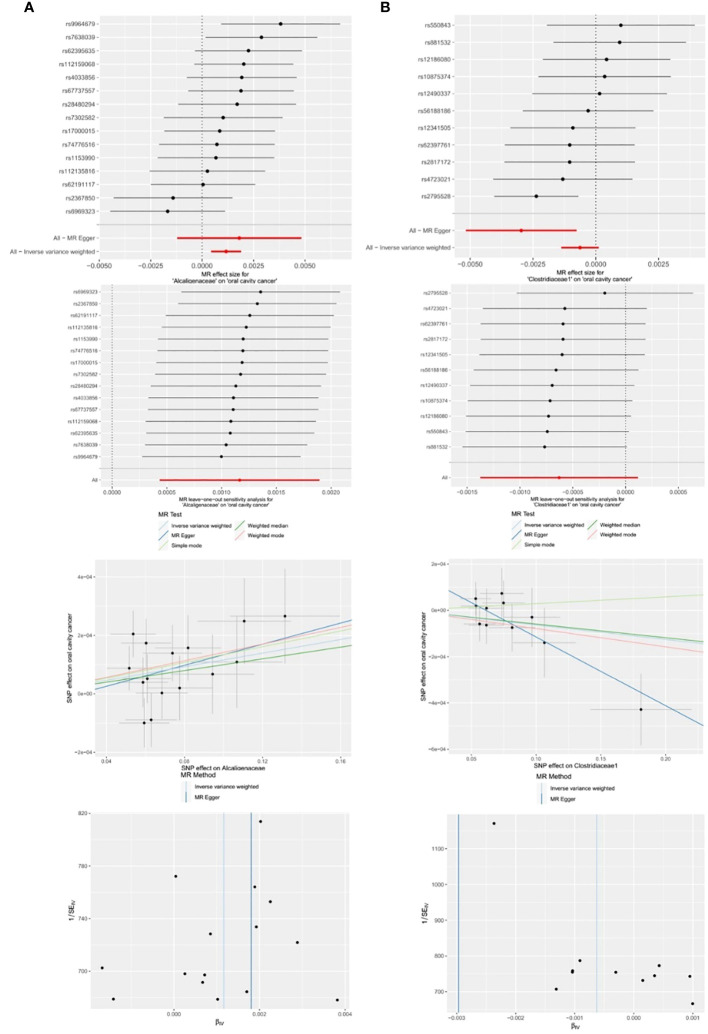
Significant MR estimates of family-level gut microbiota **(A)** *Alcaligenaceae* **(B)** *Clostridiaceae1* on OCC. From top to bottom: forest plot; leave-one-out analysis; scatter plot; funnel plot.

Among the 14 retained and selected families (*Bacteroidaceae*, *Clostridiaceae1*, *Coriobacteriaceae*, *Pasteurellaceae*, *Peptococcaceae*, *Rhodospirillaceae*, *Ruminococcaceae*, *Veillonellaceae*, *Verrucomicrobiaceae*, *Victivallaceae*, *Alcaligenaceae*, *FamilyXI*, *Enterobacteriaceae* and *Rikenellaceae*), *Alcaligenaceae* (OR = 1.0012, 95% CI = 1.0004–1.0019, P = 0.002), *Clostridiaceae1* (OR = 0.9970, 95% CI = 0.9948–0.9992, P = 0.027) and *Victivallaceae* (OR = 0.9982, 95% CI = 0.9966–0.9998, P = 0.048) were indicated to have causality on OCC in the IVW and MR-Egger (the latter two) analyses. However, significant horizontal pleiotropy was detected in the *Victivallaceae* taxa, as the MR-Egger intercept test-derived P-value was < 0.05 (*P* = *0.039*). Detailed results are presented in **Table 1** and **Figure 6**.

#### 3.3.5 MR analyses of genus level gut microbiota with OCC

Among the 63 retained and selected genera (*Adlercreutzia*, *Akkermansia*, *Alistipes*, *Anaerofilum*, *Anaerostipes*, *Bacteroides*, *Barnesiella*, *Bilophila*, *Blautia*, *Butyricicoccus*, *Butyricimonas*, *CandidatusSoleaferrea*, *Catenibacterium*, *Clostridiuminnocuum* group, *Clostridiumsensustricto1*, *Collinsella*, *Coprobacter*, *Desulfovibrio*, *Dorea*, *Eisenbergiella*, *Enterorhabdus*, *Erysipelatoclostridium*, *Erysipelotrichaceae* UCG003, *Escherichia.Shigella*, *Eubacteriumeligens* group, *Eubacteriumfissicatena* group, *Eubacteriumruminantium* group, *Eubacteriumxylanophilum* group, *Gordonibacter*, *Haemophilus*, *Holdemanella*, *Hungatella*, *Intestinimonas*, *Lachnospira*, *Lachnospiraceae* NC2004 group, *Lachnospiraceae* UCG004, *Marvinbryantia*, *Odoribacter*, *Olsenella*, *Oscillibacter*, *Paraprevotella*, *Parasutterella*, *Peptococcus*, *Phascolarctobacterium*, *Prevotella7*, *Romboutsia*, *Ruminiclostridium5*, *Ruminiclostridium6*, *Ruminococcaceae* UCG002, UCG003, UCG013, UCG014, *Ruminococcus1*, *Ruminococcaceae* UCG004, UCG009, *Ruminococcus2*, *Ruminococcusgauvreauii* group, *Ruminococcusgnavus* group, *Sutterella*, *Terrisporobacter*, *Turicibacter*, *Veillonella*, *Victivallis*), several genera were found to have causal associations with OCC in the related analyses.

- *Clostridiumsensustricto1*: IVW (OR = 0.9987, 95% CI = 0.9980–0.9995, *P* = *0.001*); MR-Egger (OR = 0.9978, 95% CI = 0.9962–0.9995, *P* = *0.035*).
- *Desulfovibrio*: IVW (OR = 1.0008, 95% CI = 1.0001–1.0015, *P* = *0.016*).
- *Eggerthella*: IVW (OR = 0.9995, 95% CI = 0.9990–1.0000, *P* = *0.048*).
- *Erysipelatoclostridium*: MR-Egger (OR = 1.0024, 95% CI = 1.0004–1.0045, *P* = *0.036*).
- *Eubacterium fissicatena* group: IVW (OR = 1.0005, 95% CI = 1.0000–1.0009, *P* = *0.032*).
- *Holdemanella*: IVW (OR = 0.9994, 95% CI = 0.9989–0.9999, *P* = *0.018*).
- *Prevotella7*: MR-Egger (OR = 1.0030, 95% CI = 1.0007–1.0054, *P* = *0.033*).

**Figure 7:**
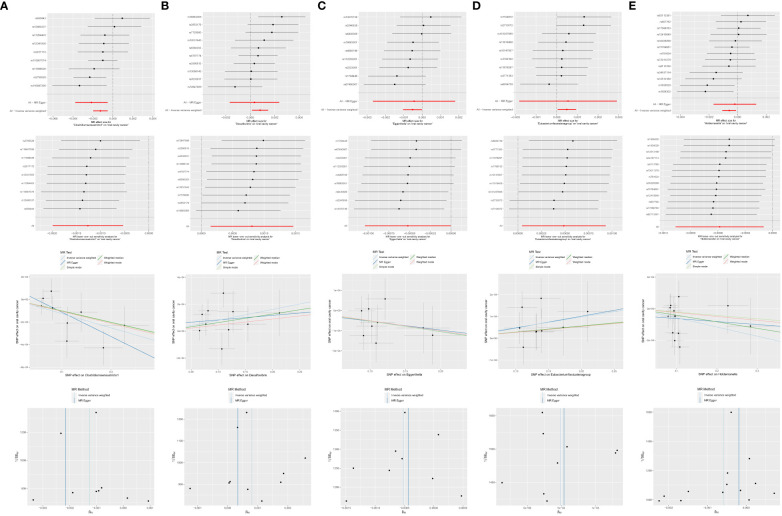
Significant MR estimates of genus-level gut microbiota **(A)** *Clostridiumsensustricto1* **(B)** *Desulfovibrio* **(C)** *Eggerthella* **(D)** *Eubacterium fissicatena* group **(E)** *Holdemanella* on OCC. From top to bottom: forest plot; leave-one-out analysis; scatter plot; funnel plot.

However, both *P* values of *Erysipelatoclostridium* (*P* = *0.020*) and *Prevotella7* (*P* = *0.038*) in MR-Egger intercept tests were <0.05, indicating significant horizontal pleiotropy. Detailed results are presented in **Table 1** and **Figure 7**.

## 4 Discussion

The multifaceted involvement of gut microbiota in the onset and progression of OCC has been suggested by a robust body of evidence from previous investigations. Despite these indications, establishing a definitive causal relationship between human gut microbiota and OCC remains challenging due to intrinsic limitations in observational studies and ethical constraints hindering experimental research in humans. In light of these challenges, our study employed a two-sample bidirectional MR method to assess the causal links between genetically predicted gut microbiota and OCC.

Our results revealed that the *Burkholderiales* order, *Alcaligenaceae* family, *Desulfovibrio* genus, and *Eubacterium fissicatena* group genus significantly elevate the risk of OCC. In contrast, the *Lentisphaerae* phylum, *Lentisphaeria* class, *Bacteroidales* order, *Victivallales* order, *Clostridiaceae1* family, *Clostridium sensu stricto1* genus, *Eggerthella* genus, and *Holdemanella* genus act as protective factors, significantly reducing the risk of OCC. These findings not only position gut microbiota as a potential marker for early identification of high-risk individuals for OCC but also open avenues for more optimized preventive and treatment strategies.

Interestingly, our study aligns with and extends the insights from three prior MR studies (19–21), collectively contributing to a more comprehensive understanding of the causal associations between gut microbiota and OCC. Notably, our research identifies additional significant risk factors (*Burkholderiales* order, *Alcaligenaceae* family, *Desulfovibrio* genus, and *Eubacterium fissicatena* group) and protective factors (*Lentisphaerae* phylum, *Lentisphaeria* class, *Bacteroidales* order, *Victivallales* order, *Clostridiaceae1* family, *Clostridium sensu stricto1* genus, *Eggerthella* genus, and *Holdemanella* genus). The observed discrepancies in results may stem from a more lenient *P* value threshold (1×10–^5^ *vs.* 5×10–^8^) for IV selection and a larger control size in the OCC GWAS data utilized.

These outcomes underscore the intricate and diverse role of gut microbiota in OCC development, emphasizing the necessity for nuanced comprehension of the microbial mechanisms involved and potential targeted interventions in OCC prevention and treatment. In coherence with the burgeoning evidence supporting a gut-oral axis, our results lay the groundwork for future investigations aimed at unraveling underlying mechanisms and identifying therapeutic targets for OCC prevention and management.

Our study not only reinforces prior findings but also introduces novel insights, particularly regarding the *Eubacterium fissicatena* group and *Desulfovibrio*, offering potential avenues for future research and clinical applications. Simultaneously, our results affirm the potential protective role of certain taxa, such as *Clostridiumsensustricto1*, in mitigating the risk of OCC. This nuanced understanding of the intricate interplay between gut microbiota and OCC enhances the prospects for targeted interventions and personalized approaches in the context of OCC prevention and treatment.

Consistent with Zhang et al. (21), we noted complete identity in original data for microbial features falling under the same taxonomic classification (*Lentisphaerae* phylum - *Lentisphaeria* class - *Victivallales* order), resulting in identical OR values (**Table 1**). This convergence may stem from characteristic dominant species within the same taxonomic classification, leading to congruence in data between upper and lower taxonomic levels. To avoid redundancy, we chose to represent data at lower taxonomic levels and refrained from analyzing similar microbial features as independent exposures. For instance, *Victivallales* order was selected from (*Lentisphaerae* phylum - *Lentisphaeria* class - *Victivallales* order). Additionally, we observed that original data for microbial features under the same taxonomic classification (*Clostridiaceae1* family - *Clostridiumsensustricto1* genus) were closely aligned, yielding approximate OR values (**Table 1**). Despite this proximity, family *Clostridiaceae* segregates into the model genus *Clostridium*, other genera, and numerous unclassified *Clostridiaceae* (totaling 49 “children” with NCBI Taxonomy ID: 31979). Moreover, while *Clostridiaceae1* demonstrated causality on OCC (OR = 0.9970, 95% CI = 0.9948–0.9992, *P* = *0.027*) in the MR-Egger analysis, *Clostridiumsensustricto1* exhibited causality as well (IVW, OR = 0.9987, 95% CI = 0.9980–0.9995, *P* = *0.001*; MR-Egger, OR = 0.9978, 95% CI = 0.9962–0.9995, *P* = *0.035*) in related analyses. Given their distinct scrutiny and the inability of *Clostridiumsensustricto1* to represent the entire *Clostridiaceae* family, retaining both taxonomic levels is justified for future studies on the causal association between gut microbiota and OCC.

In the study of Liu et al. (19), they identified *Erysipelatoclostridium* genus as a causal factor in initiating OCC risk. In our research, *Erysipelatoclostridium* also demonstrated causality on OCC (OR = 1.0024, 95% CI = 1.0004–1.0045, *P* = *0.036*) in MR-Egger analyses. However, a significant horizontal pleiotropy was observed in our research with a *P* value of *0.020* (P < 0.05) for *Erysipelatoclostridium* in MR-Egger intercept tests. Consequently, *Erysipelatoclostridium* genus was excluded. This exclusion is pivotal, underscoring the importance of addressing potential confounding factors and biases in MR studies. Horizontal pleiotropy, where a genetic variant affects multiple traits, can introduce bias in estimating causal effects if not properly addressed. Our findings suggest that while *Erysipelatoclostridium* may be associated with OCC, the observed connection might result from other factors influencing both the microbiota and disease risk, rather than a direct causal link. To further explore the role of gut microbiota in OCC, additional analyses were conducted on bacterial taxa showing less evidence of pleiotropy. These results revealed several genera consistently associated with OCC risk, offering a nuanced understanding of complex interactions between the gut microbiome and oral cancer development. These findings merit further investigation, particularly through prospective and mechanistic studies to unveil underlying biological pathways.

In conclusion, our study contributes to the growing evidence linking the gut microbiome to OCC. However, it underscores the need for cautious interpretation of MR findings and the exclusion of taxa with significant pleiotropy. Future research should aim to replicate these findings and explore potential targeted interventions to modulate the gut microbiota as a novel strategy for OCC prevention and treatment.

This study holds paramount clinical significance as it identifies specific bacterial taxa associated with OCC risk, providing a foundation for innovative interventions in the prevention and treatment of OCC by targeting the human gut microbiota. Notably, the exploration of oral probiotics/prebiotics emerges as a promising avenue. Recent evidence suggests that supplementing with oral probiotics/prebiotics can modulate gut microbiota composition, enhance immune responses, and mitigate inflammation—key factors in cancer development (36, 37). By fostering a healthy gut microbiome, these interventions may reduce OCC risk and enhance patient outcomes (38). Further research is imperative to elucidate the mechanisms underlying the effects of oral probiotics/prebiotics and establish standardized protocols for clinical use. This may pave the way for novel therapeutic strategies targeting the gut microbiome in OCC prevention and treatment, promising advancements in personalized medicine and patient care. Our study identified several taxa, including the orders of *Bacteroidales* and *Victivallales*, the *Clostridiaceae1* family, and the genera of *Clostridiumsensustricto1*, *Eggerthella*, and *Holdemanella*, demonstrating significant protective effects on OCC. These findings align with previous research (39, 40), offering potential applications in the development of new probiotics/prebiotics for individuals at risk.

Moreover, fecal microbiota transplant (FMT) emerges as another promising approach. The human gut, hosting around 1,000 bacterial species, plays a pivotal role in nutrition, immunity, and metabolism. Gut microbiome dysbiosis, characterized by an imbalance in microbial ecosystems, has been linked to various cancers, influencing cancer therapies, especially immunotherapy. FMT, involving the transfer of healthy gut microbiota from donors to patients, has shown clinical efficacy against conditions like Clostridium difficile infection (CDI) and holds promise in cancer management (41). It has the potential to reconstruct intestinal microbiota, improve bile acid metabolism, and modulate immunotherapy efficacy (42). FMT may enhance the response to immunotherapy and reduce complications, as demonstrated in patients with metastatic melanoma after anti-PD-1 therapy (43). Recognizing cancer as an ecological system has expanded therapeutic possibilities, including manipulating the gut microbiota to enhance cancer outcomes. The microbiota is now acknowledged as an emerging hallmark of cancer, influencing tumorigenesis, treatment efficacy, and toxicity (44). In our review, we emphasize FMT’s potential as a cancer therapeutic, particularly in the context of immunotherapy. This perspective opens new avenues for therapeutic interventions, offering a unique approach to improve outcomes for OCC individuals by identifying an optimal gut microbiota signature and establishing FMT feasibility in attenuating or even reversing disease progression.

While our study has primarily focused on the role of bacterial populations within the gut microbiota in relation to OCC, it is important to acknowledge the broader ecosystem of the gut. The gut microbiota is a complex and dynamic community that is predominantly composed of bacteria but also includes archaea, viruses, fungi, and even parasites, each contributing to the overall health of the host (10). Archaea, though less abundant than bacteria, have been found to play a role in various metabolic processes and may influence the composition of the bacterial community. Similarly, the viruses can impact the gut microbiota by infecting and altering bacterial populations, potentially affecting host health and disease susceptibility. Fungi, are known to interact with bacteria in a variety of ways, including competition for nutrients and space, and through the production of secondary metabolites that can inhibit bacterial growth. These interactions may have indirect effects on the bacterial populations studied in our research. Parasites, although not the main focus of our investigation, can also modulate the gut microbiota by causing dysbiosis, which may lead to alterations in the microbial balance that could influence cancer development. In the context of our findings, it is plausible that the interplay between these different components of the gut microbiota could have influenced the observed associations between specific bacterial taxa and OCC. For instance, the presence of certain fungi or the activity of viral elements might affect the stability or function of bacterial communities in ways that either promote or protect against OCC. Future research is needed to disentangle the complex interactions between bacteria, archaea, fungi, viruses, and parasites within the gut ecosystem and to elucidate their combined effects on OCC and other health outcomes. Incorporating a more holistic view of the gut microbiota, including its non-bacterial constituents, will be crucial for a comprehensive understanding of its role in health and disease. Despite the valuable insights gained from our study, certain limitations warrant consideration. Firstly, the gut microbiota-related GWAS data incorporated 18,340 participants of diverse ethnicities, while the OCC GWAS summary statistics exclusively comprised data from individuals of European descent. Although this incongruence was acknowledged, we opted for this approach based on several considerations: a) the European subset constituted the majority (nearly 80%) within the former GWAS dataset; b) this dataset boasted the largest sample size and encompassed the most comprehensive array of bacterial taxa among currently available gut microbiota-related GWAS data; c) its application in prior convincing MR studies underscored its reliability. Secondly, the original gut microbiota study conducted meta-analyses at five taxonomic levels—phylum, class, order, family, and genus—resulting in a lack of GWAS summary statistics at the species level. Consequently, our study couldn’t establish causal relationships between specific microbial species and OCC, hindering a more precise identification of an optimal gut microbiota signature. Thirdly, the nominally significant taxa identified in our analysis should be approached with caution. Although the P values from both the IVW and MR-Egger methods were below 0.05, they did not withstand the Benjamini-Hochberg correction. Consequently, these findings necessitate further validation in future studies to ascertain their definitive role, if any, in the context of OCC.

## 5 Conclusion

In summary, our study employs a comprehensive analysis to investigate the causal relationship between genetically predicted gut microbiota at various taxonomic levels and the risk of oral cavity cancer (OCC) using the two-sample bidirectional Mendelian randomization (MR) method. The results underscore the significant causal impact of *Burkholderiales* order, *Alcaligenaceae* family, *Desulfovibrio* genus, and *Eubacterium fissicatena* group in elevating OCC risk. Conversely, the *Bacteroidales* order, *Victivallales* order, *Clostridiaceae1* family, *Clostridiumsensustricto1* genus, *Eggerthella* genus, and *Holdemanella* genus emerge as causal factors associated with a decreased OCC risk. These findings not only contribute valuable insights into the understanding of OCC etiology but also pave the way for innovative interventions. Strategies such as oral administration of probiotics/prebiotics and fecal microbiota transplantation (FMT) hold promise in restoring a healthy gut microbiota and, consequently, reducing the risk of OCC. However, the quest for an optimal gut microbiota composition requires further exploration, and the intricate mechanisms governing the role of specific bacterial taxa in OCC pathophysiology warrant in-depth investigation in future studies.

## Data availability statement

The original contributions presented in the study are included in the article/supplementary material. Further inquiries can be directed to the corresponding authors.

## Ethics statement

Ethical approval was not required for the study involving humans in accordance with the local legislation and institutional requirements. Written informed consent to participate in this study was not required from the participants or the participants’ legal guardians/next of kin in accordance with the national legislation and the institutional requirements.

## Author contributions

ZS: Software, Writing – original draft, Writing – review & editing. CB: Data curation, Resources, Writing – review & editing. DH: Resources, Visualization, Writing – review & editing. XJ: Conceptualization, Funding acquisition, Project administration, Supervision, Writing – review & editing. JC: Conceptualization, Methodology, Software, Supervision, Validation, Writing – original draft, Writing – review & editing.

## References

[1] Diwan P Nirwan M Bahuguna M Kumari S Wahlang J Gupta R. Evaluating Alterations of the Oral Microbiome and Its Link to Oral Cancer among Betel Quid Chewers: Prospecting Reversal through Probiotic Intervention. Pathogens. (2023) 12:996. doi: 10.3390/pathogens12080996 37623956 PMC10459687

[2] Nien H Wang L Liao L Lin P Wu C Shueng P. Advances in image-guided radiotherapy in the treatment of oral cavity cancer. Cancers. (2022) 14:4630. doi: 10.3390/cancers14194630 36230553 PMC9561985

[3] Tang K Menezes L Baeten K Walsh L Whitfield B Batstone M. Oral HPV16 prevalence in oral potentially Malignant disorders and oral cavity cancers. Biomolecules. (2020) 10:223. doi: 10.3390/biom10020223 32028653 PMC7072384

[4] Muller S Tilakaratne W. Update from the 5th edition of the world health organization classification of head and neck tumors: tumours of the oral cavity and mobile tongue. Head Neck Pathol. (2022) 16:54–62. doi: 10.1007/s12105-021-01402-9 35312982 PMC9018914

[5] Aggarwal N Kitano S Puah G Kittelmann S Hwang I Chang M. Microbiome and human health: current understanding, engineering, and enabling technologies. Chem Rev. (2022) 123:31–72. doi: 10.1021/acs.chemrev.2c00431 36317983 PMC9837825

[6] Fan Y Pedersen O. Gut microbiota in human metabolic health and disease. Nat Rev Microbiol. (2021) 19:55–71. doi: 10.1038/s41579-020-0433-9 32887946

[7] Witkowski M Weeks T Hazen S. Gut microbiota and cardiovascular disease. Circ Res. (2020) 127:553–70. doi: 10.1161/CIRCRESAHA.120.316242 PMC7416843 32762536

[8] Ma Q Xing C Long W Wang H Liu Q Wang R. Impact of microbiota on central nervous system and neurological diseases: the gut-brain axis. J Neuroinflamm. (2019) 16:53. doi: 10.1186/s12974-019-1434-3 PMC6397457 30823925

[9] Rasouli-Saravani A Jahankhani K Moradi S Gorgani M Shafaghat Z Mirsanei Z. Role of microbiota short-chain fatty acids in the pathogenesis of autoimmune diseases. BioMed Pharmacother. (2023) 162:114620. doi: 10.1016/j.biopha.2023.114620 37004324

[10] Lozupone C Stombaugh J Gordon J Jansson J Knight R. Diversity, stability and resilience of the human gut microbiota. Nature. (2012) 489:220–30. doi: 10.1038/nature11550 PMC3577372 22972295

[11] Chen S Su T Zhang Y Lee A He J Ge Q. *Fusobacterium nucleatum* promotes colorectal cancer metastasis by modulating *KRT7-AS*/KRT7. Gut Microbes. (2020) 11:511–25. doi: 10.1080/19490976.2019.1695494 PMC7524269 31910722

[12] Wei W Li J Liu F Wu M Xiong K He Q. Alteration of intestinal microecology by oral antibiotics promotes oral squamous cell carcinoma development. Mol Immunol. (2022) 149:94–106. doi: 10.1016/j.molimm.2022.06.013 35803000

[13] Qian Y Deng C Xu X Chen Y Hu Y Hong L. Clinical analysis of immune function and intestinal flora changes in patients with oral squamous carcinoma before and after treatment. Shanghai Kou Qiang Yi Xue. (2022) 31:523–9. doi: 10.19439/j.sjos.2022.05.014 36758602

[14] Davey Smith G Hemani G. Mendelian randomization: genetic anchors for causal inference in epidemiological studies. Hum Mol Genet. (2014) 23:R89–98. doi: 10.1093/hmg/ddu328 PMC4170722 25064373

[15] Bowden J Holmes M. Meta-analysis and Mendelian randomization: A review. Res Synth Methods. (2019) 10:486–96. doi: 10.1002/jrsm.1346 PMC6973275 30861319

[16] Smith G Ebrahim S. Mendelian randomization: prospects, potentials, and limitations. Int J Epidemiol. (2004) 33:30–42. doi: 10.1093/ije/dyh132 15075143

[17] Zuccolo L Holmes M. Commentary: Mendelian randomization-inspired causal inference in the absence of genetic data. Int J Epidemiol. (2017) 46:962–5. doi: 10.1093/ije/dyw327 28025256

[18] Kurilshikov A Medina-Gomez C Bacigalupe R Radjabzadeh D Wang J Demirkan A. Large-scale association analyses identify host factors influencing human gut microbiome composition. Nat Genet. (2021) 53:156–65. doi: 10.1038/s41588-020-00763-1 PMC8515199 33462485

[19] Liu X Li X Xie M Guo J Zheng X Shi S. Association of gut microbiome and oral cavity cancer: A two sample mendelian randomization and case-control study. J Stomatol Oral Maxillofac Surg. (2023) 125:101736. doi: 10.1016/j.jormas.2023.101736 38086473

[20] Xiang K Li CX Chen R Zhao C. Genetically predicted gut microbiome and risk of oral cancer. Cancer Causes Control. (2024) 35:429–35. doi: 10.1007/s10552-023-01800-0 37815646

[21] Zhang Q Wang H Tian Y Li J Xin Y Jiang X. Mendelian randomization analysis to investigate the gut microbiome in oral and oropharyngeal cancer. Front Cell Infect Microbiol. (2024) 13:1210807. doi: 10.3389/fcimb.2023.1210807 38239501 PMC10794669

[22] Wang J Kurilshikov A Radjabzadeh D Turpin W Croitoru K Bonder MJ. Meta-analysis of human genome-microbiome association studies: the MiBioGen consortium initiative. Microbiome. (2018) 6:101. doi: 10.1186/s40168-018-0479-3 29880062 PMC5992867

[23] Brown K Godovannyi A Ma C Zhang Y Ahmadi-Vand Z Dai C. Prolonged antibiotic treatment induces a diabetogenic intestinal microbiome that accelerates diabetes in NOD mice. ISME J. (2016) 10:321–32. doi: 10.1038/ismej.2015.114 PMC4737925 26274050

[24] Luo M Sun M Wang T Zhang S Song X Liu X. Gut microbiota and type 1 diabetes: a two-sample bidirectional Mendelian randomization study. Front Cell Infect Microbiol. (2023) 13:1163898. doi: 10.3389/fcimb.2023.1163898 37313342 PMC10258312

[25] Davies N Holmes M Davey Smith G. Reading Mendelian randomisation studies: a guide, glossary, and checklist for clinicians. BMJ. (2018) 362:k601. doi: 10.1136/bmj.k601 30002074 PMC6041728

[26] Burgess S Butterworth A Thompson S. Mendelian randomization analysis with multiple genetic variants using summarized data. Genet Epidemiol. (2013) 37:658–65. doi: 10.1002/gepi.21758 PMC4377079 24114802

[27] Sanna S van Zuydam N Mahajan A Kurilshikov A Vich Vila A Võsa U. Causal relationships among the gut microbiome, short-chain fatty acids and metabolic diseases. Nat Genet. (2019) 51:600–5. doi: 10.1038/s41588-019-0350-x PMC6441384 30778224

[28] Verbanck M Chen C Neale B Do R. Detection of widespread horizontal pleiotropy in causal relationships inferred from mendelian randomization between complex traits and diseases. Nat Genet. (2018) 50:693–8. doi: 10.1038/s41588-018-0099-7 PMC6083837 29686387

[29] Bowden J Davey S Burgess S. Mendelian randomization with invalid instruments: effect estimation and bias detection through egger regression. Int J Epidemiol. (2015) 44:512–25. doi: 10.1093/ije/dyv080 PMC4469799 26050253

[30] Bowden J Davey S Haycock P Burgess S. Consistent estimation in mendelian randomization with some invalid instruments using a weighted median estimator. Genet Epidemiol. (2016) 40:304–14. doi: 10.1002/gepi.21965 PMC4849733 27061298

[31] Bowden J Del Greco M Minelli C Davey S Sheehan N Thompson J. A framework for the investigation of pleiotropy in two-sample summary data mendelian randomization. Stat Med. (2017) 36:1783–802. doi: 10.1002/sim.7221 PMC5434863 28114746

[32] Jiang M Ren L Chen S Li G. Serum uric acid levels and risk of eight site-specific cancers: A mendelian randomization study. Front Genet. (2021) 12:608311. doi: 10.3389/fgene.2021.608311 33767728 PMC7985250

[33] Hartwig F Davey S Bowden J. Robust inference in summary data mendelian randomization via the zero modal pleiotropy assumption. Int J Epidemiol. (2017) 46:1985–98. doi: 10.1093/ije/dyx102 PMC5837715 29040600

[34] Skrivankova VW Richmond RC Woolf BAR Yarmolinsky J Davies NM Swanson SA. Strengthening the reporting of observational studies in epidemiology using mendelian randomization: the STROBE-MR statement. JAMA. (2021) 326:1614–21. doi: 10.1001/jama.2021.18236 34698778

[35] Pierce B Ahsan H Vanderweele T. Power and instrument strength requirements for mendelian randomization studies using multiple genetic variants. Int J Epidemiol. (2011) 40:740–52. doi: 10.1093/ije/dyq151 PMC3147064 20813862

[36] Rajpoot M Sharma A Sharma A Gupta G. Understanding the microbiome: Emerging biomarkers for exploiting the microbiota for personalized medicine against cancer. Semin Cancer Biol. (2018) 52:1–8. doi: 10.1016/j.semcancer.2018.02.003 29425888

[37] Mohd Fuad A Amran N Nasruddin N Burhanudin N Dashper S Arzmi M. The mechanisms of probiotics, prebiotics, synbiotics, and postbiotics in oral cancer management. Probio Antimicrob Proteins. (2023) 15:1298–311. doi: 10.1007/s12602-022-09985-7 PMC9434094 36048406

[38] Khazaei Y Basi A Fernandez M Foudazi H Bagherzadeh R Shidfar F. The effects of synbiotics supplementation on reducing chemotherapy-induced side effects in women with breast cancer: a randomized placebo-controlled double-blind clinical trial. BMC Complement Med Ther. (2023) 23:339. doi: 10.1186/s12906-023-04165-8 37752516 PMC10521476

[39] Shang W Zhang S Qian H Huang S Li H Liu J. Gut microbiota and sepsis and sepsis-related death: a Mendelian randomization investigation. Front Immunol. (2024) 15:1266230. doi: 10.3389/fimmu.2024.1266230 38361921 PMC10867964

[40] Zhou X Ruan W Wang T Liu H Du L Huang J. Exploring the impact of gut microbiota on abdominal aortic aneurysm risk through a bidirectional Mendelian randomization analysis. J Vasc Surg. (2023) 30. doi: 10.1016/j.jvs.2023.11.041 38042512

[41] Zhang J Wu K Shi C Li G. Cancer immunotherapy: fecal microbiota transplantation brings light. Curr Treat Opt Oncol. (2022) 23:1777–92. doi: 10.1007/s11864-022-01027-2 PMC9589549 36279081

[42] Chen D Wu J Jin D Wang B Cao H. Fecal microbiota transplantation in cancer management: Current status and perspectives. Int J Cancer. (2019) 145:2021–31. doi: 10.1002/ijc.32003 PMC6767494 30458058

[43] Li X. Fecal microbiota transplant boosts cancer immunotherapy in patients. Am J Transplant. (2021) 21:1355. doi: 10.1111/ajt.16559

[44] Stoff R Wolf Y Boursi B. Fecal microbiota transplantation as a cancer therapeutic. Cancer J. (2023) 29:102–8. doi: 10.1097/PPO.0000000000000651 36957981

